# Mechanistic Insight into the Enhanced Anti-Pulmonary Hypertension Efficacy of Wogonin Co-Amorphous

**DOI:** 10.3390/pharmaceutics17060724

**Published:** 2025-05-30

**Authors:** Zhongshui Xie, Yucai Chen, Jiaqi Xie, Yan Lei, Chunxue Jia, Yulu Liang, Hongjuan Wang, Jianmei Huang

**Affiliations:** 1 School of Chinese Materia Medica, Beijing University of Chinese Medicine, Beijing 100102, China; 2 School of Traditional Chinese Medicine, Beijing University of Chinese Medicine, Beijing 100102, China; maxchenyucai@163.com

**Keywords:** wogonin, solubility, co-amorphous, oxidative stress, pulmonary hypertension

## Abstract

**Background:** Pulmonary hypertension (PH) remains a life-threatening rare disease characterized by inflammation and oxidative stress in pulmonary artery smooth muscle cells (PASMCs). Wogonin (Wog), a plant-derived polyphenolic compound extracted from *Scutellaria baicalensis* Georgi, exhibits notable antioxidant activity and anti-PH efficacy, whereas its clinical applications are greatly limited by poor aqueous solubility. **Methods**: Herein, an innovative wogonin-aloperine co-amorphous (Wog-Alop) was developed to improve the aqueous solubility and, thus, anti-PH efficacy of Wog. **Results**: As expected, the aqueous solubility of Wog-Alop is about 40-fold that of Wog; meanwhile, the Wog-Alop demonstrates better oral bioavailability and anti-PH efficacy than Wog; moreover, the Wog-Alop exhibits significantly enhanced capacity to attenuate oxidative stress in human PASMCs compared to Wog. **Conclusions**: The results suggested that Wog-Alop could not only improve the solubility of Wog, thereby enhancing its oral bioavailability but also alleviate Wog’s oxidative stress effects. These synergistic effects ultimately culminate in the enhanced anti-PH efficacy of Wog. In summary, the present study developed an innovative co-amorphous strategy for the delivery of Wog and improved its anti-PH efficacy.

## 1. Introduction

Pulmonary hypertension (PH) is a rare and etiologically diverse disease with an unacceptably high 5-year mortality rate [1], which is characterized by irreversible vascular remodeling and ultimately culminates in right heart failure and mortality. The pathogenesis of PH is mainly characterized by inflammation, oxidative stress, abnormal proliferation of pulmonary artery smooth muscle cells (PASMCs), and endothelial cell dysfunction [2,3]. Specifically, a growing number of studies have shown that oxidative stress contributes to the development and progression of PH through various mechanisms, including pulmonary vascular remodeling [4,5], pulmonary endothelial cell dysfunction [6,7], PASMC proliferation [8,9], and right ventricular hypertrophy [10,11]. Current anti-PH therapies include prostacyclin analogs and receptor agonists, phosphodiesterase 5 inhibitors, endothelin-receptor antagonists, and cGMP activators. Despite the improvement in quality of life, the absence of definitive curative therapies underscores the need for further research [12].

Owning to the advantages of “multi-component, multi-pathway, and multi-target”, traditional Chinese medicine plays a unique role in the treatment of complex and chronic diseases. *Scutellaria baicalensis* Georgi, also known as Chinese skullcap, has been widely used as a cardiovascular protective agent in traditional Chinese medicine. Wogonin (Wog), one of the bioactive compounds extracted from the root of *Scutellaria baicalensis* Georgi (Chinese skullcap) [13,14], exhibits significant anti-inflammatory and antioxidant properties. Wog has been reported to alleviate oxidative stress by reducing reactive oxygen species (ROS) production and malondialdehyde (MDA) levels and increasing superoxide dismutase (SOD) and catalase (CAT) activities [15,16,17]. In addition, our previous studies have demonstrated that Wog exerts an excellent inhibitory effect on platelet-derived growth factor-BB (PDGF-BB)-induced proliferation, migration, and phenotypic transformation of pulmonary artery smooth muscle cells, demonstrating notable anti-PH efficacy [18]. However, its druggability and clinical efficacy were greatly limited by poor aqueous solubility [19,20] since low solubility is often accompanied by poor bioavailability. Hence, it is crucial to improve the solubility of wogonin for the management of PH and other cardiovascular diseases.

To date, several established and emerging strategies have been addressed to enhance the solubility of wogonin, which mainly includes chemical modifications and dosage form reformulations [21,22,23]. For instance, Bian et al. [24] introduced groups at positions 7 and 8 of wogonin to synthesize its derivatives, which improved the bioavailability of wogonin and pharmacological activity but suffered from the problems of poor selectivity, low yields, and by-products [25,26]; Yang et al. [27] encapsulated wogonin into functionalized ferritin nanocarrier and enhanced cytotoxicity and prolonged the pharmacokinetics of wogonin in the mice bloodstream, with the disadvantages of low drug-loading capacity and encapsulation rate. Among these strategies, the amorphization of poorly water-soluble drugs has become one of the most effective approaches to improve solubility and dissolution and, thus, enhance drug bioavailability. However, amorphous solids lack the long-range order of molecular packing with higher internal energy and tend to crystallize during the manufacturing process compared to their crystalline counterparts.

The co-amorphous (CAM) delivery drug system has recently gained considerable interest in pharmaceutical fields as it provides opportunities for delivering poorly water-soluble drugs. The CAM is a single-phase system containing an active pharmaceutical ingredient (API) and co-amorphous co-former (CCF), contributing to the solubility, dissolution, physical stability, and bioavailability of API [28,29,30]. Therefore, aloperine (Alop), a lead compound with anti-PH activity [31,32,33], was selected as a co-former, and the wogonin-aloperine co-amorphous (Wog-Alop) were prepared by the liquid-assisted grinding method. Subsequently, the Wog-Alop was characterized by powder X-ray diffraction (PXRD), differential scanning calorimetry (DSC), thermogravimetric analysis (TGA), and Fourier transform infrared (FTIR) spectroscopy. The solubility and bioavailability were evaluated, and the efficacy against PH was further investigated in monocrotaline (MCT)-induced PH rats. On this basis, the anti-PH mechanism of Wog-Alop was investigated by measuring oxidative stress markers levers in PASMCs. To the best of our knowledge, current studies on CAM mainly focused on in vivo bioavailability while fewer studies have been conducted on efficacy and its action mechanisms, and our group investigated in vivo anti-PH efficacy of Wog-Alop and its possible action mechanisms based on solubility and bioavailability. In conclusion, the CCGNet-based co-amorphous could be developed as an innovative approach to improve the druggability of functional compounds derived from herbal medicines.

## 2. Materials and Methods

### 2.1. Materials and Agents

Wog, Alop, and monocrotaline (MCT) were bought from Yuanye Biotechnology Co., Ltd. (Shanghai, China). Ethyl carbamate, liquiritigenin, and monocrotaline were bought from Yuanye Biotechnology Co., Ltd. (Shanghai, China). Physiological sodium chloride solution was bought from Kelun Pharmaceutical Co., Ltd. (Chengdu, China). Methanol, acetonitrile, and formic acid with mass spectrometry grade were obtained from Thermo Fisher Scientific Co., Ltd. (Waltham, MA, USA).

### 2.2. Animals

Four-week-old male SPF Sprague-Dawley rats (weighing 220 ± 10 g) were purchased from Sporford Biotechnology Co., Ltd. (Beijing, China). and were randomly divided into cages (four rats per cage) and acclimatized for at least three days with free access to food and water. This study was performed with the approval of the Ethics Review Committee for Laboratory Animals of Beijing University of Chinese Medicine (approval number: BUCM-2023032307-1148) and strictly adhered to the relevant guidelines. Laboratory animal license No.: SCXK (Jing) 2019-0010; Laboratory unit use license No.: SYXK (Jing) 2020-0033.

### 2.3. Preparation of Wogonin-Aloperine Co-Amorphous

Wogonin-aloperine co-amorphous (Wog-Alop) was prepared using the liquid-assisted grinding method. Namely, wogonin (28.64 mg) and aloperine (23.23 mg) in a 1:1 molar ratio were weighed and put into a mortar and pestle, followed by adding 200 μL ultrapure water and grinding manually for 15 min. Subsequently, the mortar was placed into a drying oven at 100 °C for 10 min, and the dried powder was collected as wogonin CAM.

### 2.4. Characterization of Wogonin-Aloperine Co-Amorphous

The PXRD profiles of wogonin and Wog-Alop were carried out using Cu-Kα radiation with an operating voltage of 40 kV, a current of 150 mA, and a scanning range of 2θ: 3–80° at room temperature. Data were analyzed using Jade 6.0 software. Samples were heated in an aluminum crucible, and DSC analysis of wogonin and Wog-Alop was performed at a heating rate of 10 K/min over a temperature range of 30–350 °C using 50 mL/min nitrogen as a purge gas. FTIR spectroscopy measured scans ranging from 4000 to 650 cm^−1^ with a resolution of 4 cm^−1^ and 16 scans with a force gauge value of 80 at room temperature, 25 °C. On this basis, the stability of Wog-Alop was also investigated according to the *Pharmacopoeia of the People’s Republic of China* 2020. The Wog-Alop powder was stored at high temperature (60 °C ± 3 °C), high humidity (ambient temperature 25 °C, relative humidity 90 ± 5%), and illumination (4500 lx ± 500 lx) for 10 days, followed by PXRD analysis.

### 2.5. In Vitro Dissolution

The wogonin, physical mixture of wogonin and aloperine (Wog+Alop), and Wog-Alop with the theoretical drug content of 6.00 mg were accurately weighed and placed into 200 mL conical flasks containing 90 mL of distilled water at 37.0 °C and 100 rpm. A total of 2 mL of sample solution was withdrawn at predetermined time points (0, 1, 2, 3, 4, 5, 6, 7, 8, 9, 10, 15, 30, 45, and 60 min) and an equal volume of distilled water was immediately supplemented. The sample solutions were filtered through a 0.22 μm microporous membrane and determined by Ultra Performance Liquid Chromatography (UPLC).

### 2.6. Pharmacokinetic Studies

Twenty-four SD rats were randomly divided into three groups of eight rats each. After 3 days of adaptive feeding, they were fasted (free access to water) for 12 h. On the morning of the fourth day, wogonin, Wog+Alop, and Wog-Alop were intragastrically administered at a theoretical dose of 5 mg/kg for wogonin. Plasma samples were collected into heparinized tubes from the oculi choroideae vein at 0, 5, 10, 15, 30, 45, and 60 min and 2, 4, 6, 8, 12, 24, 32, and 48 h after administration. Plasma samples were separated by centrifugation and stored at −80 °C until determination. Ultra-Performance Liquid Chromatography-Tandem Triple Quadrupole Mass Spectrometry (UPLC-QQQ-MS) multi-response monitoring (MRM) mode was used to determine plasma samples, and Masslynx 4.1 was used to calculate plasma drug concentrations at each collection time point. The plasma concentration versus time plots were drawn, and the pharmacokinetic parameters were calculated by DAS 2.1 software. The relative oral bioavailability (RB) was calculated using the following formula:

$$\mathrm{RB}=\frac{{\mathrm{AUC}}_{\mathrm{Wog}}}{{\mathrm{AUC}}_{\mathrm{Wog}-\mathrm{Alop}}}\times 100\%$$

### 2.7. Anti-Pulmonary Hypertension Pharmacodynamics

Anti-PH pharmacodynamics were carried out using monocrotaline (MCT)-induced PH rats. Thirty rats were randomly divided into five groups: (A) control group; (B) model group; and three treatment groups (C) Wog group; (D) Wog+Alop group; and (E) Wog-Alop group. MCT was administered by a single femoral vein injection to induce PH. All administered groups of drugs were intragastrically administered from days 1 to 21 at a theoretical dose of 5 mg/kg/day for Wog. After three weeks of administration, the rats were weighed and anesthetized with 20% urethane. The electrocardiogram and right ventricular systolic pressure (RVSP) of rats were measured. The rats were perfused, and the heart and lung tissues were completely removed. The right ventricle (RV) and left ventricle plus ventricular septum (LV + S) were accurately separated and weighted, and the right ventricular hypertrophy index (RVHI) = RV/(LV + S) × 100%, RV mass index (RVMI) = RV/body weight × 100% and lung mass index (LMI) = lung weight/body weight × 100% were calculated. Right ventricular and left lung tissues were fixed in 4% formalin solution for 48 h, embedded in paraffin, and sectioned. Heart tissues were taken for H&E and Masson staining, and lung tissues were stained with resorcinol basic fuchsin and proliferating cell nuclear antigen (PCNA) immunofluorescence.

### 2.8. Cell Culture and Treatment

Human PASMCs (HPASMCs, Cat. No.: CYH0707) were purchased from Wuhan Enji Life Technology Co., Ltd. (Wuhan, China) HPASMCs were cultured in Dulbecco’s Modified Eagle Medium (DMEM) medium with 10% fetal bovine serum (FBS) and 1% penicillin/streptomycin (P/S), and maintained in a humidified 5% CO_2_ incubator at 37 °C. Prior to drug treatment, HPASMCs were subjected to serum starvation by culturing in a maintenance medium containing 0.5% FBS for 24 h to synchronize cell cycle progression. For the experiments, cells were pretreated with a blank medium or 3 μM of Wog, Alop, Wog+Alop, and Wog-Alop, respectively. After 1 h, cells were stimulated with 40 ng/mL PDGF-BB for 48 h in each group except the control group.

### 2.9. Cell Viability Assay

Cell viability was assessed using the Cell Counting Kit-8 (CCK-8) assay according to the manufacturer’s protocol. Briefly, 10 μL of CCK-8 reagent was added to each well of pretreated PASMCs, followed by incubation at 37 °C under 5% CO_2_ for 2-h. The absorbance of each sample was quantified at 450 nm using a microplate reader.

### 2.10. ROS Detection

To investigate the anti-oxidative effects of Wog-Alop on PASMCs under oxidative stress, PDGF-BB-induced PASMCs were treated with different drugs for 48 h. After treatment, cells were loaded with 1 μM 2′,7′-dichlorodihydrofluorescein diacetate (DCFH-DA) probe for 20 min at 37 °C in the dark. Nuclei were counterstained with Hoechst 33342 Live Cells Staining Solution (1:100 dilution) for 10 min. Fluorescent imaging was performed using a laser scanning confocal microscope (Leica TCS SP8, Wetzlar, Germany) with excitation/emission settings at 488/525 nm for DCFH-DA and 350/461 nm for Hoechst 33342. ROS-associated fluorescence intensity was quantified using ImageJ 4.4 software.

### 2.11. Measurement of Oxidative Stress Markers

The levels of MDA and glutathione (GSH), as well as the activities of CAT and glutathione peroxidase (GPx) in cells, were measured using commercial assay kits. The treated PASMCs were collected and lysed with protein lysis buffer following the manufacturer’s instructions.

### 2.12. Statistical Analysis

Data were shown as the mean ± SD. Statistical analysis was performed via GraphPad Prism 9 (GraphPad Software; LaJoya, CA, USA). Statistical comparisons were made using one-way ANOVA followed by Dunnett’s post hoc test. Significance was defined as *p* < 0.05.

## 3. Results

### 3.1. Preparation and Characterization of Wog-Alop

**Figure 1:**
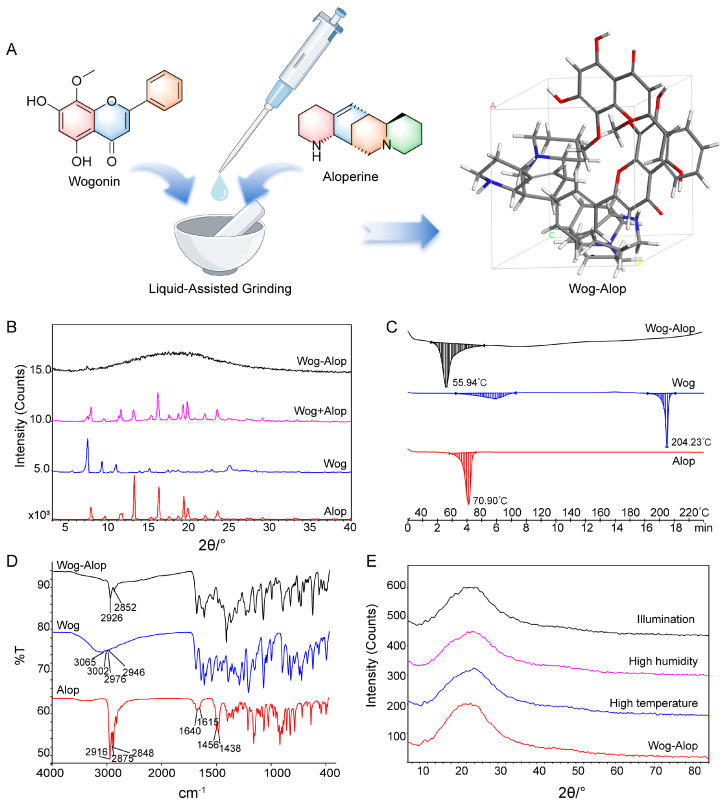
Preparation and characterization of Wog-Alop. (**A**) Preparation process of Wog-Alop; (**B**) PXRD patterns of wogonin, aloperine, Wog+Alop and Wog-Alop; (**C**) DSC patterns of wogonin, aloperine and Wog-Alop; (**D**) FTIR patterns wogonin, aloperine and Wog-Alop; (**E**) Stability of Wog-Alop in illumination, high humidity, high temperature (Alop: aloperine; Wog: wogonin; Wog+Alop: the physical mixture of wogonin and aloperine; Wog-Alop: Wogonin-aloperine co-amorphous).

The Wog-Alop was obtained by liquid-assisted grinding [34,35] with a 1:1 molar ratio of wogonin and aloperine (Figure 1A). The powder X-ray diffraction (PXRD), differential scanning calorimetry, and Fourier transform infrared (FTIR) spectroscopy were used to confirm the formation of Wog-Alop. As shown in Figure 1B, a broad hump (“amorphous halo”) [36] was observed in Wog-Alop while the diffraction peaks of wogonin and aloperine disappeared. The DSC thermogram revealed that enthalpy relaxation masking the glass transition of Wog-Alop was observed at 56 °C, which differs from Wog (88 °C and 204 °C) and Alop (71 °C), further indicating the formation of Wog-Alop, (Figure 1C). Moreover, the characteristic O-H stretching vibration frequency of wogonin at 3200 cm^−1^ and the characteristic stretching frequency -NH of aloperine at 1600 cm^−1^ disappeared (Figure 1D), respectively, which could be speculated that there was a strong hydrogen bond interaction between wogonin and aloperine, indicating that the new lattice arrangement emerged in Wog-Alop. In addition, the stability Wog-Alop were investigated (Figure 1E), and the PXRD patterns of Wog-Alop after 10 days at high temperature, high humidity and illumination were consistent with the initial patterns, implying satisfactory stability of Wog-Alop.

### 3.2. In Vitro Dissolution and In Vivo Pharmacokinetics of Wog-Alop

**Figure 2:**
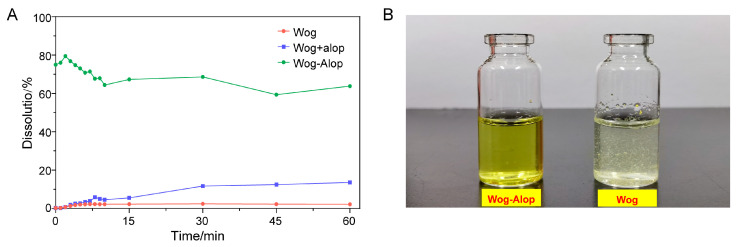
In vitro dissolution (**A**) and solubility states (**B**) of Wog-Alop in water.

**Figure 3:**
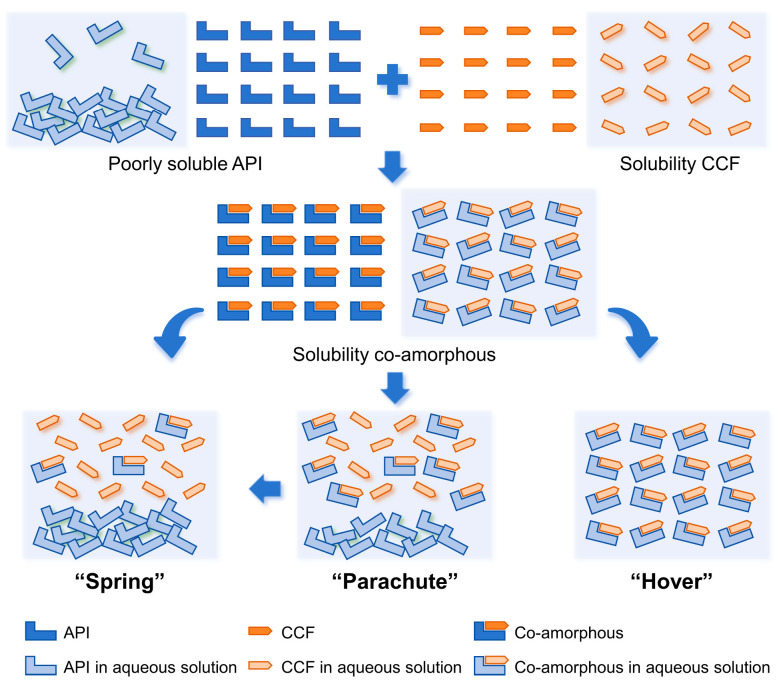
The dissolving process of insoluble drugs in aqueous environment.

The in vitro dissolution of wogonin, Wog+Alop, and Wog-Alop were investigated; the dissolution behaviors are shown in Figure 2A,B. The percentage of dissolution of wogonin was only 2.17%, and the improved solubility (13.59%) was observed in Wog+Alop due to the addition of aloperine. Interestingly, Wog-Alop rapidly dissolves and reaches the supersaturated state in water, leading to a 40-fold increase in equilibrium dissolution percentage compared to wogonin. In other words, Wog-Alop could maintain constant high dissolution behavior similar to the “spring-hover” effect, attributed to the suitable CCF, which can effectively inhibit the solution-mediated re-crystallization (Figure 3) [37,38].

**Figure 4:**
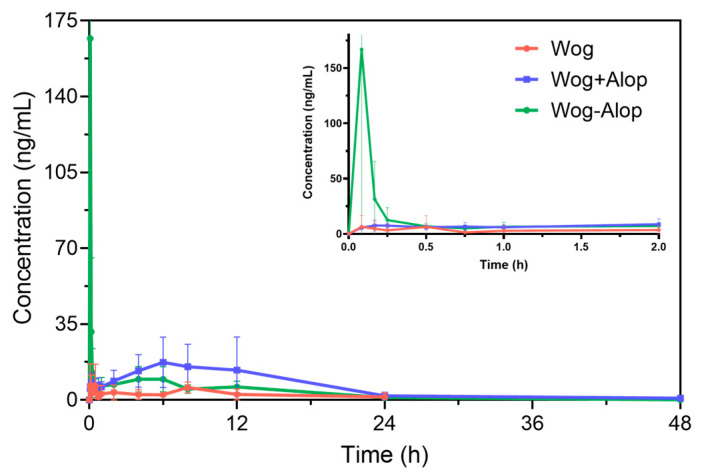
In vivo pharmacokinetics of Wog-Alop.

Accordingly, the Wog-Alop was rapidly absorbed in vivo with a *C*_max_ of 262.93 ± 159.26 ng/mL (Figure 4 and Table 1), which is 17.82-fold higher than that of the wogonin. The relative bioavailability of Wog-Alop was elevated to 218.683%, indicating that Wog-Alop could improve the oral bioavailability of wogonin by improving the solubility [39].

### 3.3. In Vivo Anti-Pulmonary Hypertension Pharmacodynamics

#### 3.3.1. Hemodynamics and Myocardial Electrophysiology

**Figure 5:**
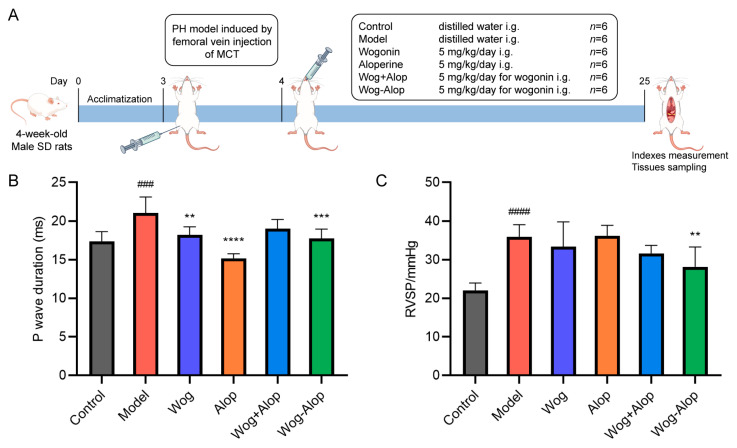
Effect of Wog-Alop on hemodynamics and myocardial electrophysiology of rats from each group. (**A**) Schematic diagram of in vivo anti-PH efficacy experiments; (**B**) Effect of Wog-Alop on P-wave duration; (**C**) Effect of Wog-Alop on RVSP. Data are expressed as the mean ± SD, n = 6, ^###^ *p* < 0.001 vs. control group, ^####^ *p* < 0.0001 vs. control group; **** *p* < 0.0001 vs. model group, *** *p* < 0.001 vs. model group, ** *p* < 0.01 vs. model group.

The efficacy of Wog-Alop against PH was further investigated by monocrotaline (MCT)-induced PH rats (Figure 5A). The results of myocardial electrophysiology showed that the P-wave duration of MCT-PH was significantly prolonged (*p* < 0.001). Wog (*p* < 0.01), Alop (*p* < 0.0001), and Wog-Alop (*p* < 0.001) treatments could all significantly reduce the P-wave duration of MCT-PH rats (Figure 5B). The hemodynamic results showed that the RVSP of MCT-PH rats was significantly increased. Compared to the model group, the Wog and Wog+Alop groups showed reduced RVSP, although without statistical significance (*p* > 0.001). In contrast, the Wog-Alop group exhibited a significant decrease in RVSP (*p* < 0.05, Figure 5C).

#### 3.3.2. Right Ventricular Remodeling

**Figure 6:**
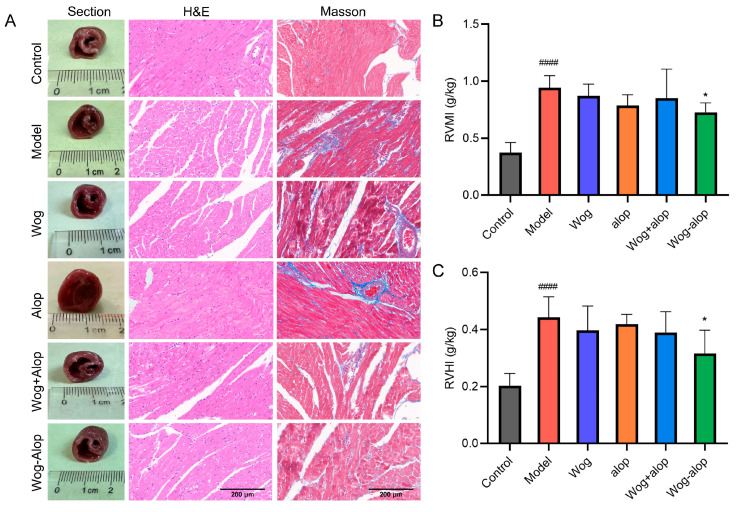
Effect of Wog-Alop on the right ventricular remodeling of rats from each group. (**A**) Effect of Wog-Alop on right ventricular remodeling (40×); (**B**) Effect of Wog-Alop on RVMI; (**C**) Effect of Wog-Alop on RVHI. Data are expressed as the mean ± SD, n = 6, ^####^ *p* < 0.0001 vs. control group; * *p* < 0.05 vs. model group.

RVMI and RVHI evaluated the effect of Wog-Alop on right ventricular hypertrophy in MCT-PH rats. The results showed that Wog-Alop treatment could significantly reduce the RVMI and RVHI of MCT-PH rats (*p* < 0.05). Although Wog, Alop, and Wog+Alop could reduce RVMI and RVHI, the differences were not statistically significant (*p* > 0.05, Figure 6B,C). Furthermore, morphological analysis of ventricular cross-sections revealed that Wog-Alop had a significant improvement effect on right ventricular hypertrophy caused by MCT-PH. Pathological injury of myocardial tissue and myocardial fibrosis were evaluated by HE staining and Masson staining (Figure 6A). The results indicated that myocardial tissue in MCT-PH rats exhibited a disorganized, disordered, and fragmented arrangement, with significant inflammatory cell infiltration in the right ventricular myocardium and severe myocardial fibrosis. All drug-treated groups showed varying degrees of improvement in inflammatory infiltration and fibrosis, among which the Wog-Alop group demonstrated the most pronounced therapeutic efficacy.

#### 3.3.3. Pulmonary Vascular Remodeling

**Figure 7:**
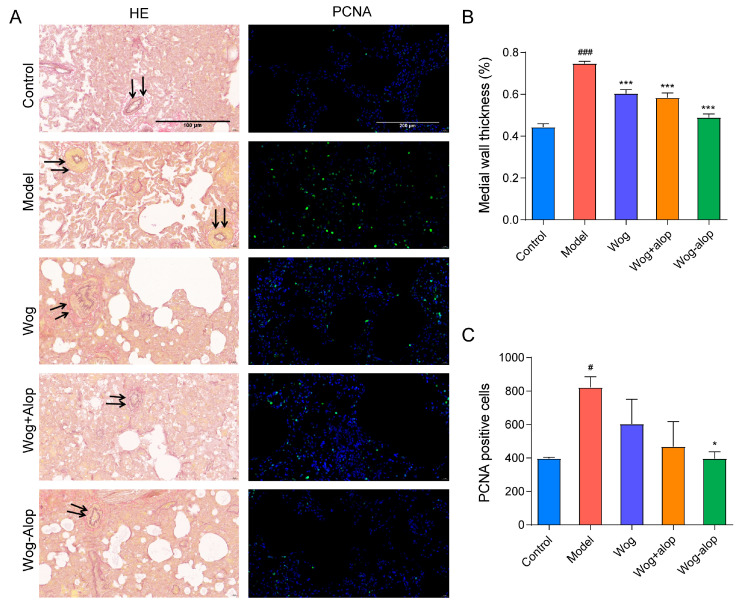
Effect of Wog-Alop on pulmonary vascular remodeling of rats from each group. (**A**) Resorcinol basic fuchsin staining and PCNA immunofluorescence of lung tissues; (**B**) Effect of Wog-Alop on the medial wall thickness of small pulmonary artery vessels; (**C**) Effect of Wog-Alop on PCNA positive cells of lung tissue. Data are expressed as the mean ± SD, *n* = 6, ^###^ *p* < 0.001 vs. control group, ^#^ *p* < 0.05 vs. control group, *** *p* < 0.001 vs. model group, * *p* < 0.05 vs. model group.

In order to better study the effect of Wog-Alop on pulmonary vascular remodeling, the thickening of the medial wall of pulmonary vascular and the PCNA immunofluorescence of rat lung tissue sections were assayed (Figure 7A). As shown in Figure 7B, all administration groups could inhibit the thickening of the medial wall of small pulmonary artery vessels to varying degrees (*p* < 0.001), in the order of Wog-Alop group > Wog+Alop group > wogonin group. In addition, the abnormal PCNA proliferation could be reduced by administration groups, and only the Wog-Alop group indicates a statistical difference (*p* < 0.05, Figure 7C). These results demonstrated the significant advantage of CAM in improving oral bioavailability and efficacy.

### 3.4. Cell Culture and Treatment

**Figure 8:**
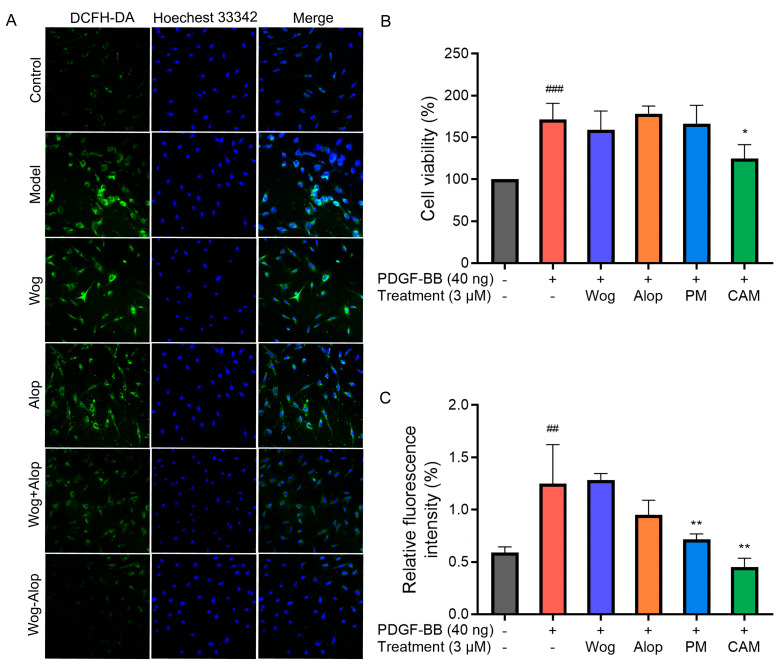
Effects of different drug treatments on abnormal proliferation and ROS in PDGF-BB-induced PASMCs. (**A**) Representative images of ROS levels in PDGF-BB-induced PASMCs; (**B**) Effects of 3 μM Wog, Alop, Wog+Alop (PM), and Wog-Alop (CAM) treatments on the abnormal proliferation of PDGF-BB-induced PASMCs; (**C**) Effects of 3 μM Wog, Alop, Wog+Alop (PM), and Wog-Alop (CAM) treatments on ROS levels in PDGF-BB-induced PASMCs (*n* = 3, ^##^ *p* < 0.01 vs. Control group, ^###^ *p* < 0.001 vs. Control group; * *p* < 0.05, ** *p* < 0.01 vs. Model group).

The inhibitory effects of Wog, Alop, Wog+Alop (PM), and Wog-Alop (CAM) on the abnormal proliferation of PDGF-BB-induced PASMCs were investigated at a concentration of 3 μM. As shown in Figure 8B, PDGF-BB treatment significantly increased PASMC proliferation (*p* < 0.001). When the concentration of Wog was controlled at 3 μM in all treatment groups, Wog, Alop, and Wog+Alop exerted almost no inhibitory effects on abnormal proliferation of PDGF-BB-induced PASMC. In contrast, Wog-Alop (CAM) significantly inhibited abnormal proliferation of PDGF-BB-induced PASMC (*p* < 0.05). These results indicated that the formation of the Wog-Alop significantly enhanced the inhibitory activity of Wog against abnormal proliferation of PASMC.

### 3.5. ROS Detection

ROS are oxygen-containing free radicals, including hydrogen peroxide (H_2_O_2_), superoxide anion (O_2_^−^), and nitric oxide (NO). Excessive accumulation of ROS may exert adverse effects on pulmonary artery vasculature, such as promoting vasoconstriction, exacerbating endothelial injury, and triggering inflammatory responses, thereby accelerating pulmonary vascular remodeling and the progression of PH [40,41]. The effects of different drug treatments on ROS in PDGF-BB-induced PASMCs are shown in Figure 8A,C. Under normal conditions, PASMCs exhibited low ROS levels, whereas PDGF-BB stimulation significantly increased intracellular ROS (*p* < 0.001). Neither Wog nor Alop alone showed notable inhibitory effects on ROS overproduction in PDGF-BB-induced PASMCs. In contrast, both Wog+Alop (PM) and Wog-Alop (CAM) significantly reduced ROS levels in PDGF-BB-induced PASMCs (*p* < 0.01). Notably, Wog-Alop (CAM) demonstrated a stronger inhibitory effect, restoring ROS to levels comparable to those in normal cells. These results suggested that the anti-oxidative stress activity of Wog-Alop (CAM) is not merely an additive effect of Wog and Alop but may also be associated with the potential formation of a complex in the aqueous phase.

### 3.6. Measurement of Oxidative Stress Markers

MDA, the end product of lipid peroxidation, exhibits a positive correlation with mean pulmonary arterial pressure (mPAP) and disease severity in PH patients. CAT is a critical antioxidant enzyme that can catalyze the decomposition of hydrogen peroxide (H_2_O_2_) into water and oxygen, thereby mitigating ROS toxicity. Glutathione (GSH) is the primary intracellular non-enzymatic antioxidant that directly neutralizes ROS. And glutathione peroxidase (GPx) is a selenium-dependent antioxidant enzyme that utilizes GSH to reduce lipid peroxides and H_2_O_2_ into harmless products. Diminished GPx activity is directly associated with enhanced pulmonary artery constriction and right ventricular hypertrophy. Therefore, the levels of MDA and GSH, as well as the activities of CAT and GPx in PASMCs, were measured.

**Figure 9:**
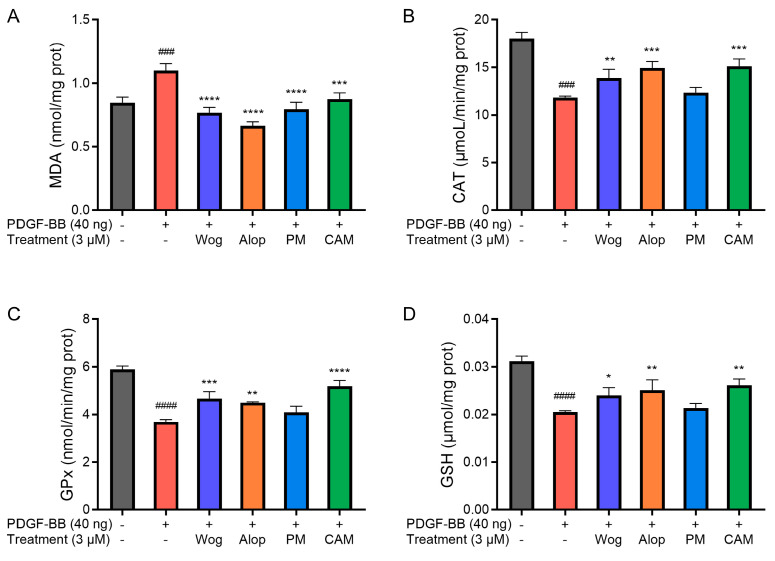
Effects of 3 μM Wog, Alop, Wog+Alop (PM), and Wog-Alop (CAM) treatments on MDA level (**A**), CAT activity (**B**), GPx activity (**C**), and GSH levels (**D**) in PDGF-BB-induced PASMCs (*n* = 3, ^###^ *p* < 0.001 vs. Control group, ^####^ *p* < 0.0001 vs. Control group; * *p* < 0.05 vs. Model group, ** *p* < 0.01 vs. Model group, *** *p* < 0.001 vs. Model group, **** *p* < 0.0001 vs. Model group).

Figure 9 demonstrates the effects of different drug treatments on oxidative stress-related parameters in PDGF-BB-induced PASMCs. As shown in the figures, a significant increase in MDA level (*p* < 0.001) and a decrease in CAT (*p* < 0.001) and GPx (*p* < 0.0001) activities, as well as in GSH level (*p* < 0.0001), have resulted from oxidative stress in PDGF-BB-induced PASMCs. The oxidative stress of PASMCs induced by PDGF-BB was alleviated to a certain extent after treatment with different drugs. Among them, the increase in MDA levels in PDGF-BB-induced PASMCs was significantly inhibited by Wog, Alop, Wog+Alop, and Wog-Alop treatments. The reduction in CAT and GPx activities and GSH levels in PDGF-BB-induced PASMCs were significantly inhibited by Wog, Alop, and Wog-Alop, and the regulation of Wog-Alop was the closest to the normal level. In summary, oxidative stress in PDGF-BB-induced PASMCs could been mitigated by Wog-Alop through modulating ROS, MDA, GSH, CAT, and GPx, thereby exerting anti-PH effects. The superior efficacy of Wog-Alop may arise from the enhanced activity conferred by a potential complex formed in the aqueous phase rather than merely reflecting the additive effects of its individual components.

## 4. Discussion

Oral administration is the main route of traditional Chinese medicine, but some functional compounds derived from herbal medicines are difficult to transport and be absorbed through the gastrointestinal tract due to low solubility, poor permeability, efflux of transit proteins, and intestinal metabolism, among others, affecting their druggability and clinical efficacy [42]. The CAM drug delivery system has emerged as a promising approach for the improvement of insoluble functional compounds, which not only exhibits considerable advantages in enhancing solubility and physical stability but also provides a strategy for combination therapy of clinical applications. Herein, an innovative Wog-Alop was successfully synthesized, which demonstrated improved solubility and better anti-PH efficacy than wogonin. Furthermore, Wog-Alop demonstrated superior efficacy compared to Wog in reducing ROS and MDA levels while enhancing CAT and GPx activities in PDGF-BB-induced PASMCs. These results suggested that Wog-Alop could not only improve the solubility of Wog, thereby enhancing its oral bioavailability but also amplify Wog’s anti-oxidative stress effects. These synergistic effects ultimately enhance Wog’s therapeutic potential against PH. Nevertheless, considerable challenges in developing CAM remain to be addressed. For instance, a large number of in vivo studies are required to study the pharmacological activities and the potential role of CCF in physiological environments intensively. What’s more, the scale-up preparation and downstream processing of the CAM formulations into final dose forms remain a challenging issue [43]. Despite these challenges, CAM has proven to be a very promising technique to improve the solubility and druggability of functional compounds derived from herbal medicines.

## 5. Conclusions

In the present study, the oral bioavailability and anti-PH activity of Wog were significantly enhanced by preparing the Wog-Alop co-amorphous formulation. This remarkable improvement could be attributed to strategic conformational modifications in the Wog molecule induced by the co-amorphization process, which synergistically elevated its solubility and amplified its antioxidant efficacy. Collectively, this work provides a practical framework for repurposing insoluble natural products into clinically applicable therapies for PH and diverse oxidative stress-mediated pathologies.

## Figures and Tables

**Table 1:** Pharmacokinetic parameters ($\bar{x}$ ± s, *n* = 8).

Parameter	Wogonin	Wog+Alop	Wog-Alop
AUC_(0–*t*)_ (ng·h/mL)	49.119 ± 42.102	142.467 ± 70.458 **	133.233 ± 41.228 **
AUC_(0–∞)_ (ng·h/mL)	76.999 ± 80.556	148.573 ± 67.134 *	168.384 ± 61.471 *
MRT_(0–*t*)_ (h)	5.968 ± 4.792	7.491 ± 2.079	6.265 ± 2.336
MRT_(0–∞)_ (h)	12.502 ± 17.461	11.341 ± 5.585	11.363 ± 9.549
t_1/2z_ (h)	7.212 ± 9.973	4.078 ± 2.226	7.317 ± 6.772
*T*_max_ (h)	1.933 ± 3.412	3.783 ± 3.355	0.083 ± 0
*C*_max_ (ng/mL)	14.757 ± 12.221	18.393 ± 8.135	262.933 ± 159.257 ****
RB (%)	-	192.954	218.683

* *p* < 0.05 vs. Wog. ** *p* < 0.01 vs. Wog. **** *p* < 0.0001 vs. Wog.

## Data Availability

The data used to support the findings of the study are available upon reasonable request from the corresponding author.

## Abbreviations

The following abbreviations are used in this manuscript:

PH	Pulmonary hypertension
PASMCs	Pulmonary artery smooth muscle cells
ROS	Reactive oxygen species
MDA	Malondialdehyde
SOD	Superoxide dismutase
CAT	Catalase
Wog	Wogonin
Wog+Alop	The physical mixture of wogonin and aloperine
Wog-Alop	Wogonin-aloperine co-amorphous
PDGF-BB	Platelet-derived growth factor-BB
CAM	Co-amorphous
API	Active pharmaceutical ingredient
CCF	Co-amorphous co-former
PXRD	Powder X-ray diffraction
DSC	Differential scanning calorimetry
FTIR	Fourier transform infrared
MCT	Monocrotaline
UPLC	Ultra-performance liquid chromatography
UPLC-QQQ-MS	Ultra-performance liquid chromatography-tandem triple quadrupole mass spectrometry
RVSP	Right ventricular systolic pressure
RV	Right ventricle
LV	Left ventricle
S	Ventricular septum
RVHI	Right ventricular hypertrophy index
LMI	Lung mass index
PCNA	Proliferating cell nuclear antigen
DMEM	Dulbecco’s modified Eagle medium
FBS	Fetal bovine serum
P/S	Penicillin/streptomycin
CCK-8	Cell counting kit-8
DCFH-DA	2′,7′-dichlorodihydrofluorescein diacetate
GSH	Glutathione
GPx	Glutathione peroxidase
